# Case Report: Cerebrospinal Fluid Dynamics in the Optic Nerve Subarachnoid Space and the Brain Applying Diffusion Weighted MRI in Patients With Idiopathic Intracranial Hypertension—A Pilot Study

**DOI:** 10.3389/fneur.2022.862808

**Published:** 2022-04-15

**Authors:** Jatta Berberat, Achmed Pircher, Philipp Gruber, Karl-Olof Lovblad, Luca Remonda, Hanspeter Esriel Killer

**Affiliations:** ^1^Institute of Neuroradiology, Kantonsspital Aarau, Aarau, Switzerland; ^2^Department of Psychiatry, University of Geneva, Geneva, Switzerland; ^3^Department of Neuroscience/Ophthalmology, Uppsala University, Uppsala, Sweden; ^4^Division of Diagnostic and Interventional Neuroradiology, Geneva University Hospitals and Faculty of Medicine of Geneva, Geneva, Switzerland; ^5^Faculty of Medicine, University of Bern, Bern, Switzerland; ^6^Department of Biomedicine, University of Basel, Basel, Switzerland

**Keywords:** CSF, optic nerve (ON), MRI, diffusion imaging, idiopathic intracranial hypertension (IIH), papilledema

## Abstract

**Purpose:**

The aim of this study was to examine the cerebrospinal fluid (CSF) flow rates in the subarachnoid space (SAS) of the optic nerve (ON) and the brain in patients with idiopathic intracranial hypertension (IIH) and papilledema (PE) compared to healthy controls by applying non-invasive diffusion-weighted MRI.

**Methods:**

A retrospective analysis of diffusion-weighted MR images of 5 patients with IIH (10 ONs), mean age: 31 ± 10 years (5 women), and 11 healthy controls (22 ONs, mean age: 60 ± 13 years, 5 women) was performed. The flow velocity flow-range ratio (FRR) between the intracranial cavity and the SAS of the ON was calculated in both groups and then compared.

**Results:**

The mean FRR was 0.55 ± 0.08 in patients with IIH and 0.63 ± 0.05 in healthy controls. The difference between patients with IIH and healthy controls was statistically significant (*p* < 0.05).

**Conclusions:**

The CSF flow velocity was decreased in patients with IIH with PE compared to healthy controls. The reduced CSF flow dynamics might be involved in the pathophysiology of PE in IIH and diffusion-weighted MRI can be a useful non-invasive tool to study the CSF flow dynamics within the SAS ON.

**Summary:**

Idiopathic intracranial hypertension is a neurological disease, where vision loss is the most feared complication of this disorder. The pathophysiology of IIH is not fully understood but is strongly linked to a reduced uptake of CSF into the central dural sinus veins. In this study, we examined the CSF flow rates in the SAS ON and the brain in patients with IIH and PE compared to healthy controls by applying non-invasive diffusion-weighted MRI. Knowing about the flow ratio of CSF may be of clinical relevance for the treatment decisions of IIH. If medical treatment fails, surgical options for lowering the ICP pressure need to be taken into consideration. As the primary goal of treatment is to prevent the loss of vision and visual field, it is important to know whether the communication of CSF between the intracranial CSF and the CSF in the perioptic space is intact. We showed that the CSF flow velocity was decreased in IIH patients with PE compared to healthy controls. The reduced CSF flow might be involved in the pathophysiology of PE in IIH, and diffusion-weighted MRI can be a useful non-invasive tool to study the CSF flow dynamics within the SAS ON.

## Introduction

Idiopathic intracranial hypertension (IIH) is a neurological disorder that presents with headache, loss of vision and visual field, tinnitus, and occasionally double vision. The ophthalmologic hallmark of IIH is swelling of the optic disc, papilledema (PE), which if untreated can lead to damage of the visual field, visual acuity and loss of color vision, and even legal blindness (1, 2). The cause of these disturbances is an elevated intracranial pressure (ICP) in the absence of any known causative factors (mass lesion, inflammation, central venous thrombosis).

The pathophysiology of PE is still not fully understood. The swelling of the optic disc is thought due to axon compression resulting from increased ICP that is transmitted *via* the optic canal to the subarachnoid space (SAS) of the optic nerve (ON). In a recent study, Pircher et al. demonstrated impaired contrast loaded cerebrospinal fluid (CSF) concentrations within the ON SAS in 16 patients with IIH and PE by CT cisternography (3). A reduced contrast loaded CSF is a clear indicator for impaired CSF dynamics in a compartmentalized SAS of the ON. Due to the invasive nature of CT cisternography, a non-invasive method to study the CSF flow dynamics is highly desirable.

Boye et al. have developed a methodology to calculate the flow ratios within the ON SAS and the brain by using diffusion-weighted MR images (4). In diffusion-weighted imaging (DWI), the particles moving parallel to a magnetic field gradient undergo a phase shift, proportional to their velocity. The phase shift can then be used to determine the flow rate range. In this study, the same measurement method is used to examine the flow ratio within the ON SAS in patients with IIH and PE. Due to the relationship of pressure and velocity in the Bernoulli equation, this technique may allow for calculation of intrasheath pressure.

## Methods

### Subjects

Five patients with IIH (10 ONs, mean age: 31 ± 10 years, 5 women) with IIH and PE underwent diffusion-weighted MRI. All patients underwent a neuro-ophthalmological examination prior to MRI including slit lamp-assisted biomicroscopy of the ocular fundus, testing of color vision, and visual acuity and visual field testing. Lumbar puncture and CSF examination were performed in all cases in order to establish the diagnosis that was based on the updated modified Dandy criteria (5). Clinical symptoms consisted blurry vision, transient visual obscuration, visual field disturbances, and headache. The mean body mass index (BMI) measured 33 ± 11. The mean CSF opening pressure measured by lumbar puncture was 35 ± 11 cm H_2_O.

A cohort of 11 healthy controls (22 ONs, mean age: 60 ± 13 years, 5 women) were included. No included controls had a history of neurological symptoms or ON diseases. Further, their MRI did not show any pathological findings. This retrospective study was approved by the Local Ethical Committee and conducted in accordance with the Declaration of Helsinki.

### Imaging

Images were acquired with a 3T whole body magnet (Skyra; Siemens Healthcare, Erlangen, Germany) with a 32-channel head coil using Stejskal-Tanner (6) diffusion sequence using the following parameters: b = 50 s/mm^2^, TE/TR = 65/2,000 ms, 6 slices, 1-mm slice thickness with acquisition time of 4.13 min, each slice acquired 120 times. The diffusion sequence was used to estimate the water diffusivity in MR image voxels and allowed to estimate the flow velocities of coherent moving particles through phase contrast images as described in detail in Boye et al. (4). Shortly, the monopolar diffusion gradients of the diffusion sequence lead to a constant phase shift for coherently moving particles. Using the b-values, the maximum encoded velocity (venc) before a phase wrap occurs can be solved. The phase shift can then be used to determine the flow velocity range. Since the phase shift of the diffusion sequence is highly irregular, the results are presented as flow range ratio (FRR), which allows the easy comparison between different groups.

### Statistics

A statistical analysis was performed with unpaired *t*-test performed by the SPSS Statistics Software version 21 (IBM Corporation, Armonk, NY, USA) to compare the FRR results between the patients with IIH and healthy controls.

## Results

The average FRR was 0.55 ± 0.08 in patients with IIH and.63 ± 0.05 in healthy controls. The FRR of patients with IIH ranged from 0.40 to 0.72 and in healthy controls from 0.59 to 0.68. A statistically significant difference was not observed between the right and the left ON in patients with IIH (*p* = 0.1) as well as those in the healthy controls (*p* = 0.1). When comparing the FRR of the patients with IIH and healthy controls, a statistically significant difference (*p* < 0.05) was detected. The results are summarized in Table 1.

**Table 1:** Summary of the patient parameters.

**#**	**Age**	**BMI**	**ICP [cmH_**2**_O]**	**Visual field Goldmann**	**IOP OD [mmHg]**	**IOP OS [mmHg]**	**MD OD**	**MD OS**	**FRR OD**	**FRR OS**
1	38	21	29	Concetric constriction	20	18		24	0.54	0.40
2	20	39	29		16	16			0.52	0.52
3	22	24	50	big blind spot	15	15	10.5	6.0	0.62	0.61
4	30	50	42	Inferionasal field cut	17	17	8.9	9.5	0.71	0.56
5	45	33	24		16	15	13.2	10.2	0.56	0.48

*BMI, body mass index; ICP, intracranial pressure; IOP, intraocular pressure; MD, mean defect; OD, ocula dextra; OS, ocula sinistra*.

## Discussion

Idiopathic intracranial hypertension is a neurological disease that presents with headaches, tinnitus, and PE. Vision loss is the most feared complication of this disorder (1, 2). The pathophysiology of IIH is not yet fully understood but the core issue is linked to a reduced uptake of CSF into the central dural sinus veins.

The pressure and content of CSF are considered homogenous in all CSF-containing spaces in the spine, the brain, and the ON. The concept of a continuous pressure and content of CSF, however, has been challenged by studies on CSF protein concentration gradients between lumbar CSF and perioptic CSF as well as by the results of cisternographic studies that demonstrated concentration gradients of contrast-loaded CSF between the basal cisterns and the SASs of ONs (3, 7). Clinical evidence for CSF pressure differences between the lumbar site and the SAS of the ON was reported by Kelman et al. (8). Further examples suggesting inhomogeneous pressure distributions are patients with asymmetric and unilateral PE (9–12).

A recent publication on the symmetry of PE demonstrated a possible link between the ON canal patency and the severity of PE (13). Furthermore, a study on patients with IIH demonstrated a partial compartmentation of the SAS of the ON with cisternography (3). In such cases, a free CSF pathway from the intracranial suprasellar cistern into the orbital SAS of the ON can be severely impeded. The conclusion of such studies is that CSF pressure and content can vary between different locations in the brain and the SAS of the ON.

This condition is also related to a current problem in space flight (14). A considerable number of astronauts develop the spaceflight-associated neuro-ocular syndrome (SANS) (15). Compartmentalization of the SAS ON is also currently used to explain partly the optic disc swelling in SANS (16, 17). Data about the flow rate in different CSF spaces is therefore important for the diagnosis of ON sheath compartmentation.

Knowing about the flow ratio of CSF may also be of clinical relevance for the treatment decisions of IIH. If medical treatment fails, surgical options for lowering ICP pressure need to be taken into consideration; among them are lumbo- and ventriculoperitoneal shunts and ON sheath fenestration (ONSF) (18). As the primary goal of treatment is to prevent the loss of vision and visual field, it is important to know whether the communication of CSF between the intracranial CSF and the CSF in the perioptic space is intact. If there is no indication of compartmentalisation of CSF in the perioptic space and headache is untreatable with medication, a shunt procedure is a possible treatment of choice. If, however, headache is mild and compartmentalisation can be assumed, ONSF might be a more adequate treatment, as it addresses the pathology at the site of the problem.

In order to make this distinction, CSF flow analysis can be helpful. Given a lower flow rate within the ON SAS, a pressure gradient due to compartmentation can be suspected. Unlike cisternography, flow rate determination is a non-invasive procedure.

Although the ratios we calculated in this study are not absolute values, they still can help to compare the intracranial flow rate to the flow rate in the SAS of the ON as well as between the two ONs. Given a significantly lower flow rate in the SAS of the ON compared to the intracranial flow rate, a slower flow in the SAS and therefore a higher pressure (according to Bernoulli) are to be expected. Further studies with larger groups are necessary to enhance the validity of this method.

As a conclusion, reduced CSF flow dynamics might be involved in the pathophysiology of PE in IIH, and diffusion-weighted MRI can be a useful non-invasive tool to study the CSF flow dynamics within the SAS ON.

## Data Availability Statement

The original contributions presented in the study are included in the article/supplementary material, further inquiries can be directed to the corresponding author/s.

## Ethics Statement

The studies involving human participants were reviewed and approved by PB_2017-00652. The patients/participants provided their written informed consent to participate in this study. Written informed consent was obtained from the individual(s) for the publication of any potentially identifiable images or data included in this article.

## Author Contributions

All authors listed have made a substantial, direct, and intellectual contribution to the work and approved it for publication.

## Funding

Financial support from the Swiss National Science Foundation (Grant No. 196877) is acknowledged.

## Conflict of Interest

The authors declare that the research was conducted in the absence of any commercial or financial relationships that could be construed as a potential conflict of interest.

## Publisher's Note

All claims expressed in this article are solely those of the authors and do not necessarily represent those of their affiliated organizations, or those of the publisher, the editors and the reviewers. Any product that may be evaluated in this article, or claim that may be made by its manufacturer, is not guaranteed or endorsed by the publisher.
